# Cerebral venous sinus thrombosis associated with SRA-negative heparin-induced thrombocytopenia: case report

**DOI:** 10.1186/s12959-023-00490-7

**Published:** 2023-04-21

**Authors:** Floyd D. Silva, Allison E. Burnett, Anand Padmanabhan, Marian A. Rollins-Raval, Noah P. Splinter, Masoom J. Desai

**Affiliations:** 1 University of New Mexico School of Medicine, Albuquerque, NM USA; 2 University of New Mexico College of Pharmacy, Albuquerque, NM USA; 3 Division of Experimental Pathology, Department of Laboratory Medicine and Pathology, Mayo Clinic, Rochester, MN USA; 4 Department of Pathology, University of New Mexico School of Medicine, Albuquerque, NM USA; 5 Department of Neurology, University of New Mexico School of Medicine, Albuquerque, NM USA

**Keywords:** Heparin-induced thrombocytopenia. Cerebral venous sinus thrombosis. Serotonin release assay. Case report. P-selectin expression assay. Anticoagulation. 4T score.

## Abstract

**Background:**

There are very few documented reports in literature of cerebral venous sinus thrombosis (CVST) caused by immune-mediated heparin-induced thrombocytopenia (HIT). Further, there are very few reports of false negative serotonin release assays (SRAs) when testing for immune-mediated HIT.

**Case presentation:**

We present a case of a 60- year-old male with recent unfractionated heparin administration for venous thromboembolism prophylaxis, an elevated 4T score of 5 and acute CVST in which immune-mediated HIT was suspected. The enzyme-linked immunosorbent assay (ELISA) screening assay was positive for PF4 antibodies and subsequent reflexive SRA testing was negative. However, given the clinical picture, a false-negative SRA was suspected (and eventually confirmed), prompting use of the alternative PF4-dependent p-selectin expression assay (PEA) which was confirmed to be positive. The patient was successfully managed with a bivalirudin infusion and eventually transitioned to apixaban.

**Conclusion:**

It is uncommon for immune-mediated HIT with thrombosis to manifest as CVST. Similarly, false-negative SRA is uncommon in immune-mediated HIT. Take-away lessons from our case report include considering HIT in CVST patients with an elevated 4T score and considering the entire clinical picture and degree of suspicion for HIT when interpreting negative HIT testing results. The PEA, in conjunction with the 4Ts score, may be considered as an alternate diagnostic assay for HIT.

## Background

Immune-mediated heparin-induced thrombocytopenia (HIT), an infrequent complication of heparin administration, is caused by the production of antibodies directed against heparin-platelet protein factor 4 (PF4) and is considered to be a hypercoagulable state requiring the use of a non-heparin anticoagulant [1]. The incidence of immune-mediated HIT is estimated to be 0.1-5% in patients receiving therapeutic doses of heparin. One study found that the incidence of pathologic PF4 antibody formation in patients receiving UFH and LMWH is 17% and 8%, respectively, though this number varies significantly depending on the population of interest being studied [1]. HIT-associated thromboembolism does not typically manifest as CVST but rather other manifestations, including but not limited to deep venous thrombosis, pulmonary embolism, and peripheral arterial thrombosis [2], One systematic review found the incidence of CVST in HIT to be 1.6%, with SRA considered as the gold standard in diagnosis [3]. Our case highlights the importance of considering further investigation in a CVST patient who presents with atypical initial workup: a negative serotonin-release assay (SRA), which is highly sensitive for HIT and considered to be the gold standard for confirmatory diagnosis following a positive enzyme-linked immunosorbent assay (ELISA).

## Case presentation

**Fig. 1:**
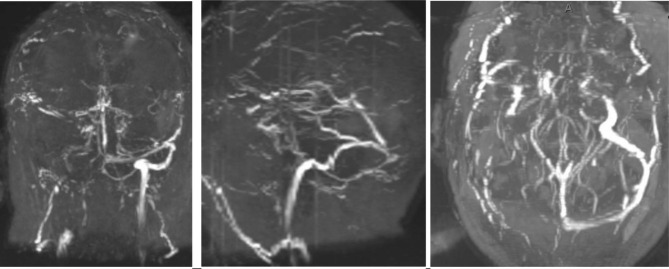
Coronal view, sagittal view, and axial view of MR Venogram of head without contrast demonstrating non-visualization of flow related signal within the super sagittal, right sigmoid and right transverse sinuses consistent with venous sinus thrombosis (Day 22)

**Fig. 2:**
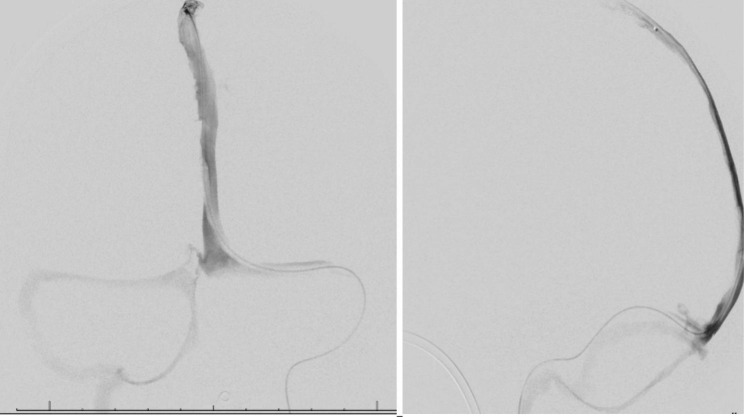
Digital Subtraction Angiogram on day 23 showing microcatheter positioned in middle 1/3rd of superior sagittal sinus and extensive occlusive clot distal to the catheter

**Fig. 3:**
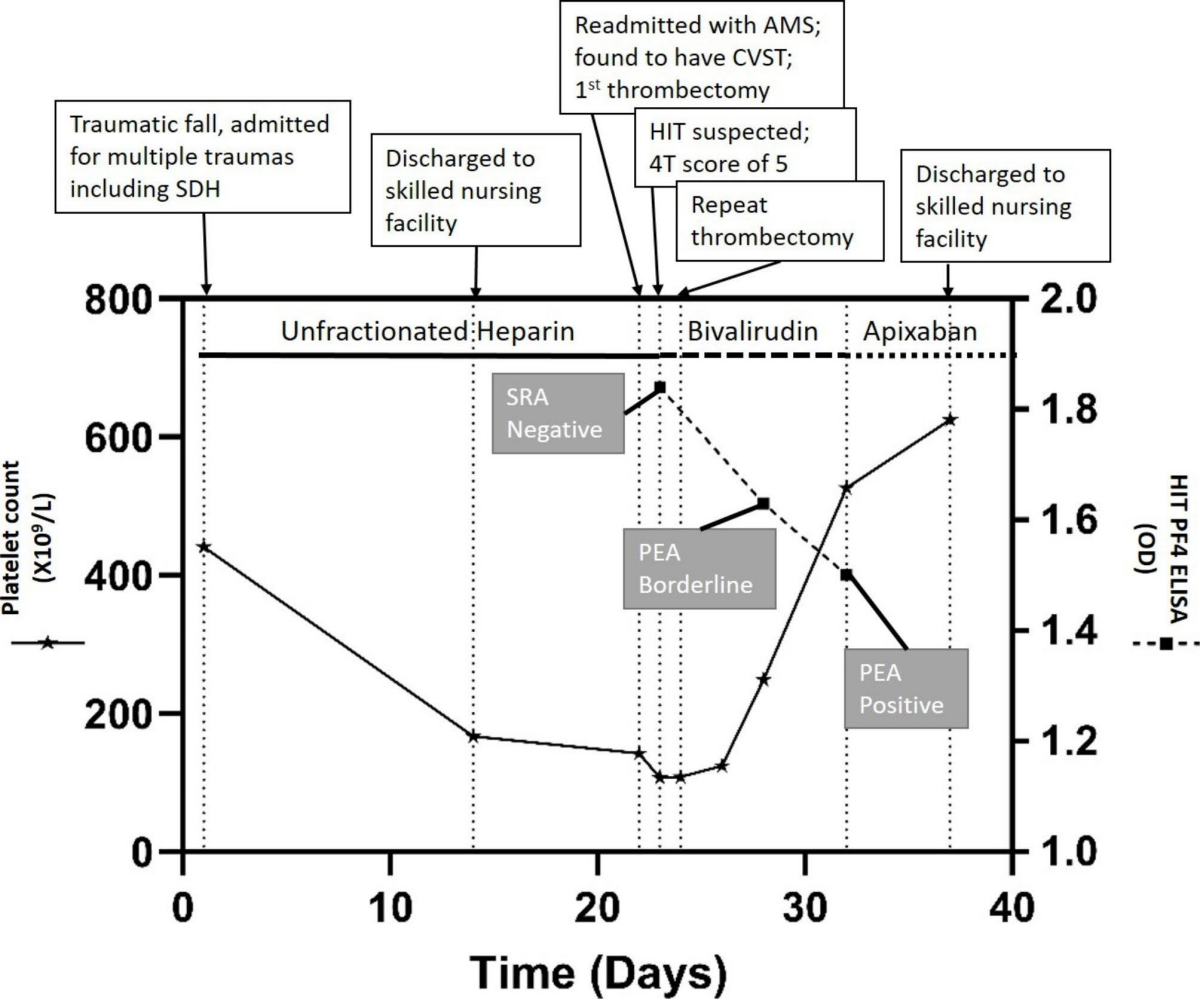
Timeline of patient clinical and laboratory findings. Unfractionated heparin at 5000U TID SubQ from Day 1–23; bivalirudin 250 mg [0.13 mg/kg/hr] + NaCl 0.9% (Infusion) from Day 23–32; Apixaban 5 mg BID from Day 32. SDH: Subdural hemorrhage; AMS: Altered mental status; CVST: cerebral venous sinus thrombosis; HIT: Heparin Induced Thrombocytopenia; SRA: serotonin release assay; PEA: P-selectin expression assay; PF4: Platelet Factor 4

A male in his 60s with a past medical history of type 2 diabetes, hypertension, tobacco, daily alcohol use, COVID vaccination without details of dosing, date, or manufacturer, and recent left-sided subdural hemorrhage due to traumatic intraparenchymal hemorrhage (day 1) presented to our emergency department for decreased mentation and difficulty with ambulation 8 days after hospital discharge (day 22). At the discharge facility, he was continued on subcutaneous unfractionated heparin for venous thromboembolism prophylaxis which had been initiated at our hospital. Upon presentation, he was readmitted for decreased mentation and difficulty ambulating due to weakness in the bilateral lower extremities. Vitals including temperature, systolic and diastolic blood pressure, pulse rate, and respiratory rate were within normal limits and the patient had oxygen saturations in the 90s on room air. Neurologic examination was significant for eye opening to noxious stimuli, following simple commands, and expressive aphasia. Significant cranial nerve findings included right gaze preference unable to cross midline, right upper and lower extremity strength 3/5, and left upper and lower extremity strength 1/5. A stroke alert was called, and MR venogram demonstrated multiple CVSTs affecting the superior sagittal, right sigmoid, and right transverse sinuses (Figs. 1 and 2) for which mechanical thrombectomy and microcatheter-directed thrombolysis with 10 mg recombinant tissue plasminogen activator (rtPA) was performed. On the second day of admission, a CT of the chest showed a right upper lobe segmental pulmonary embolism with RV/LV < 1. Duplex ultrasound was negative for DVT. Two days later, repeat Duplex showed bilateral upper extremity cephalic thrombophlebitis, for which therapeutic-intensity unfractionated heparin was initiated. Thrombocytopenia developed with a 4T score of 5. Heparin was empirically switched to bivalirudin titrated to an aPTT of 1.5-2.5x mean lab normal due to suspected HIT. Additionally, HIT PF4 IgG-specific antibody ELISA (Zymutest HIA IgG, Aniara, West Chester, OH) was sent and came back positive at 1.84 OD [Reference Interval (RI) 0.00-0.30 negative, 0.31–0.49 weakly positive, 0.50-3.00 positive]. This sample was screened with an anti-Xa assay for heparin and was negative. It was reflexively sent out for SRA (ARUP, Salt Lake City, UT) and was reported as negative with 6% serotonin release in response to low dose UH (0.1) (RI ≤ 20 serotonin release). Due to high suspicion for HIT despite the negative SRA, additional testing was sent with HIT PF4 antibody which was again positive at 1.63 OD with reflex to a P-Selectin Expression Assay (PEA) (Versiti, Milwaukee, WI). The PEA results were borderline positive with P-Selectin (PS) Expression at 31% (RI ≤ 35%) in the presence of patient serum and PF4 30 mcg. Finally, a third sample was sent with a HIT PF4 antibody of 1.58 OD with reflex to PEA which showed positive PS expression at 45% in the presence of patient serum and PF4 30 mcg with complete inhibition of PS expression in the presence of 100U/ml of heparin, confirming the diagnosis of HIT. The subtle difference in PEA results between the two samples was attributed to be likely due to donor platelet variability. Given the high incidence of CVST in patients with a newly recognized entity, vaccine-induced immune thrombotic thrombocytopenia (VITT), the patient was tested in a novel, research-based VITT specific ELISA (“un-complexed PF4” ELISA), which was negative in the second and third samples tested (0.31 and 0.32 optical density, OD; cut off 1.0 OD). Complete normalization of platelet count was observed by day 27 of admission. Patient was eventually transitioned to apixaban 5 mg PO twice a day (induction dosing had been completed with bivalirudin) for a planned duration of at least 3 months. His platelets during the previous hospital admission for the traumatic SDH ranged from 200 to 300 × 10^3^ µl, and it was confirmed that the heparin had been continued for VTE prophylaxis at the rehabilitation facility. During that time, his platelets ranged from 89 to 163 × 10^3^ µl. Thrombocytopenia resolved and patient was transitioned to apixaban. The patient was discharged to a skilled nursing facility when medically stable (Fig. 3). He was referred to outpatient hematology specialists, but he was eventually lost to follow up as he switched his care to a different hospital system.

## Discussion and conclusions

First described in 2018, SRA-negative HIT is a rare complication of heparin administration that can manifest in a number of organ systems [4]. Despite the possibility of a negative assay, one study of 430 subjects suggested that SRA remains largely sensitive in the diagnosis of HIT [5]. Workup in patients with suspected immune-mediated HIT includes evaluation of time of onset of thrombocytopenia after heparin initiation, and diagnostic criteria includes a “≥ 30% drop from baseline platelet count resulting in an absolute thrombocytopenia ≤ 150 × 109/L or even a normal platelet count” [6]. Due to significant overdiagnosis, clinicians are strongly encouraged to use the well-validated 4T pre-test clinical probability score which recommends testing in any patient with a score of ≥ 4. Our case highlights the importance of further diagnostic workup in the case of a patient presenting with multiple thromboemboli and an elevated 4T score despite a negative SRA, including HIT PF4 antibody and PEA. To summarize, as a result of this case presentation, we suggest further diagnostic evaluation in the patient who presents with high suspicion for HIT in the setting of negative SRA.

## Data Availability

Data sharing is not applicable to this article as no datasets were generated or analyzed during the current study.
